# Supplementary material for: Deep learning-based cross-classifications reveal conserved spatial behaviors within tumor histological images
Source: Nat Commun. 2020 Dec 11;11:6367. doi: 10.1038/s41467-020-20030-5 (PMC7733499; doi:10.1038/s41467-020-20030-5)
Supplement: Supplementary file 1 — Supplementary Information [file 41467_2020_20030_MOESM1_ESM.pdf]

Supplementary Figures of “Deep learning-based cross-classifications reveal conserved spatial behaviors within tumor histological images”

Javad Noorbakhsh[1*], Saman Farahmand[2*], Ali Foroughi pour[1*], Sandeep Namburi[1], Dennis Caruana[3], David Rimm[3], Mohammad Soltanieh-ha[4], Kourosh Zarringhalam[2,5], Jeffrey H. Chuang[1,6]

[1]The Jackson Laboratory for Genomic Medicine, Farmington, CT
[2]University of Massachusetts-Boston, Computational Sciences PhD program, Boston, MA.
[3]Yale University School of Medicine, Department of Pathology, New Haven, CT
[4]Boston University, Department of Information Systems, Boston, MA
[5]University of Massachusetts-Boston, Department of Mathematics, Boston, MA.
[6]UCONN Health, Department of Genetics and Genome Sciences, Farmington, CT
[*]These authors contributed equally.

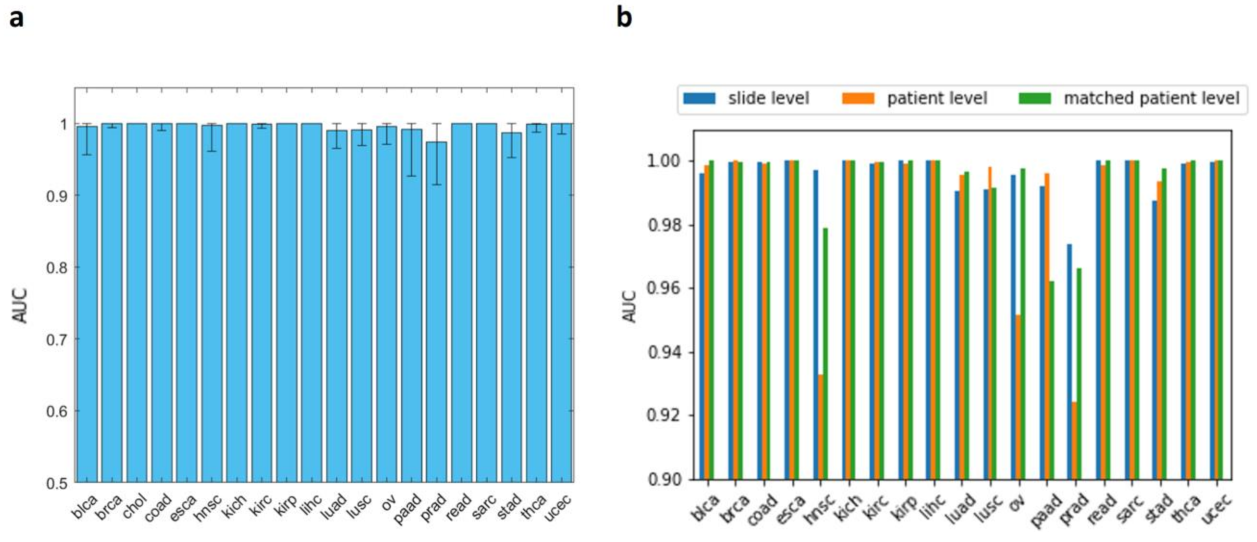

Supplementary Figure 1. (a) Per-slide AUC values for tumor/normal classifiers and their confidence intervals. The height of each bar denotes the mean AUC and error bars denote the lower and upper bounds of the CI. The cancer types with small or imbalanced test data are the ones that tend to have poorer performance. Note that a generic CI cannot be assigned to cancer types with an AUC of 100%. (b) Per-slide AUC values for tumor/normal classifiers for the slide level, patient level, and matched patient level splits of data. The difference between the patient level split and slide level split across all cancer types is -0.007±-0.02, and the difference between the matched patient level split and slide level split across all cancer types is -0.002±-0.009. TCGA slide level test set sizes are provided in Figure 2a, and patient level sample sizes are provided in Supplementary Data 5.

a

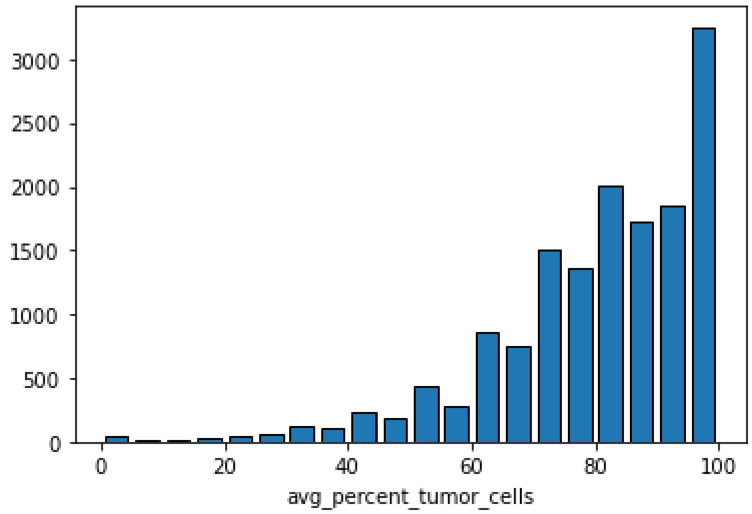

b

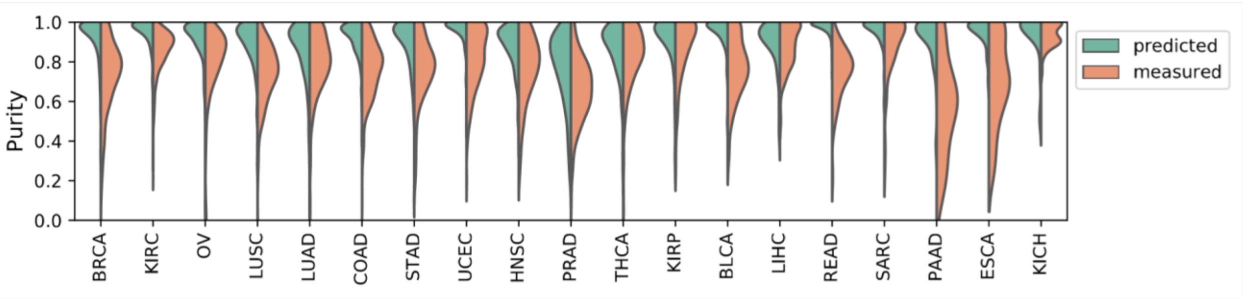

Supplementary Figure 2. (a) The distribution of purity (TCGA-annotated "average percent tumor cells") across TCGA slides. (b) Distribution of tumor purity as predicted by our CNN model, compared to the TCGA pathologist measurements. TCGA sample sizes are provided in Figure 2a.

a. TCGA-90-6837-11A-01-TS1

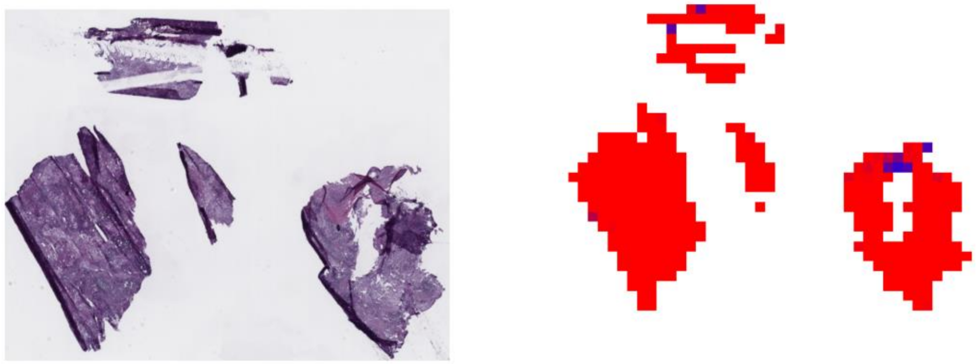

b. TCGA-04-1638-11A-01-TS1

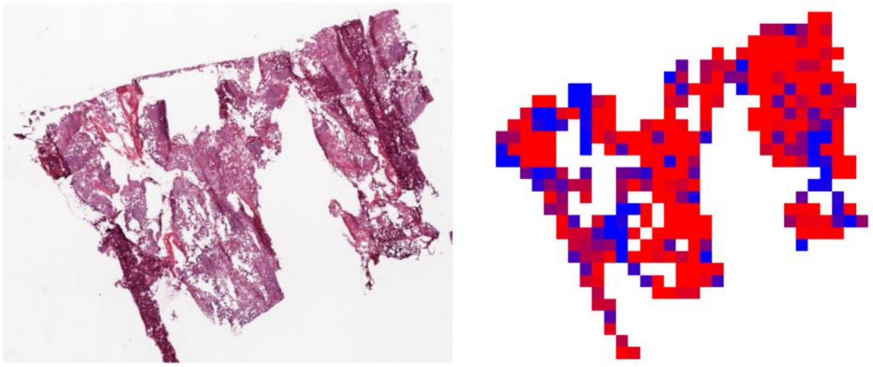

c. TCGA-AA-3655-11A-01-TS1

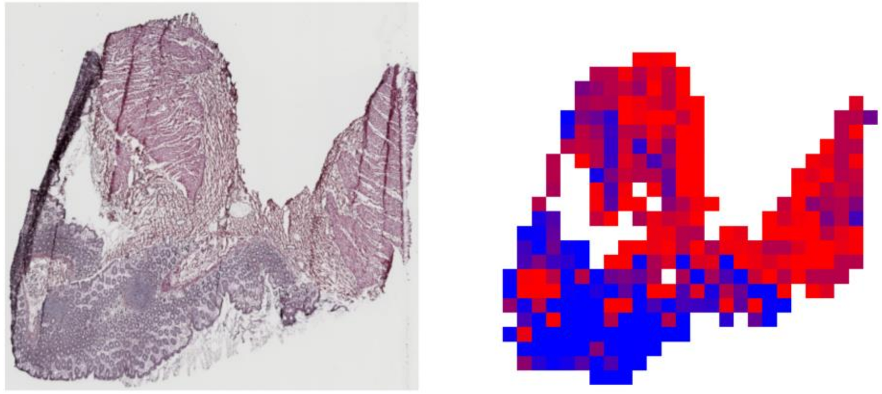

Supplementary Figure 3: Example of slides labeled as adjacent normal by TCGA but as tumor by the CNN. Manual pathology review indicates misclassified slides often suffer from poor quality, tissue folding, or excessive tissue damage related to freezing. The red and blue denote regions of high or low predicted tumor probability, respectively. (a) A LUSC adjacent normal suffering from tissue folding. (b) An OV adjacent normal with tissue folding. (c) A COAD adjacent normal where predictions appear to be impacted by freezing damage. Regions without damage are classified correctly.

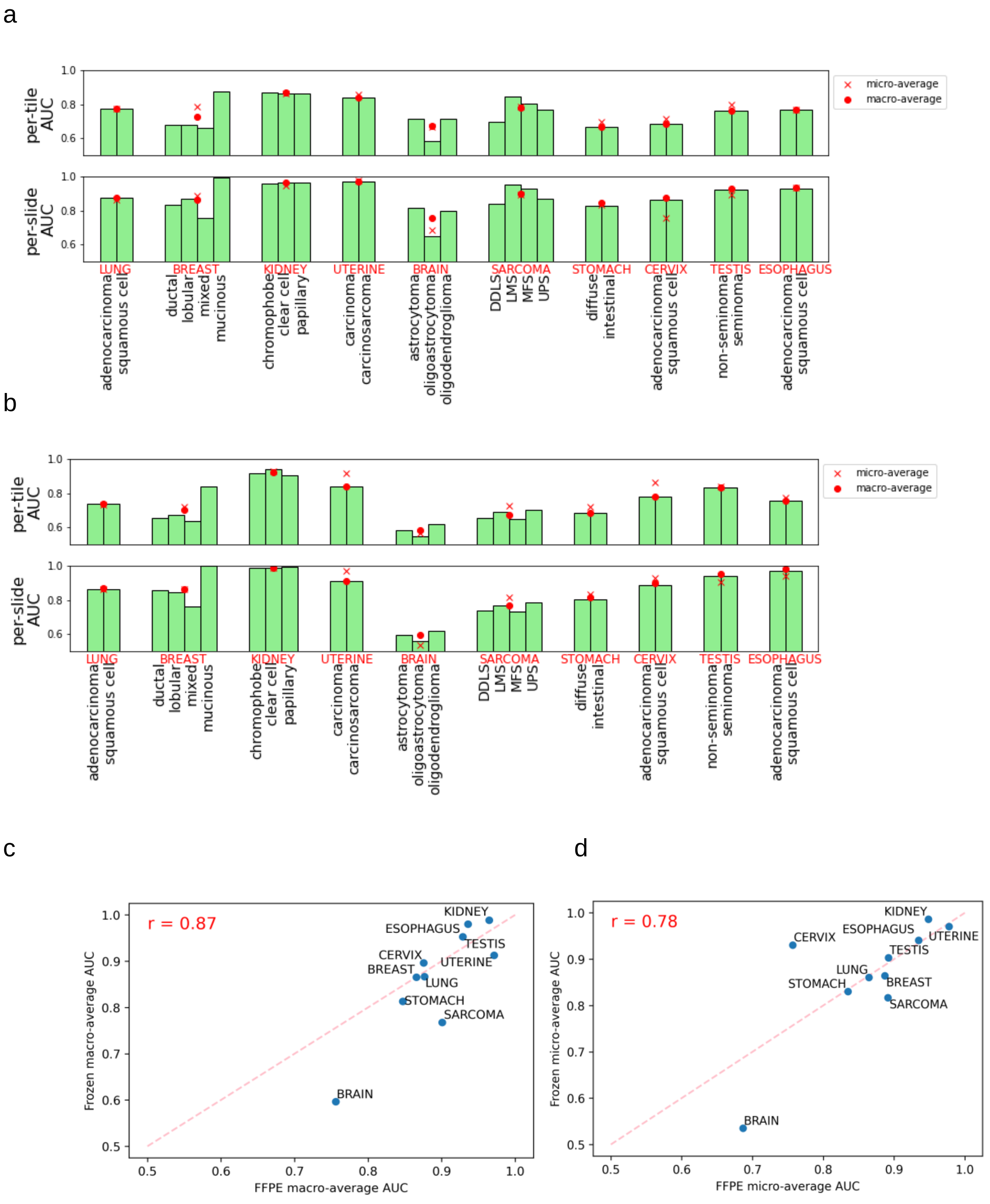

Supplementary Figure 4. Subtype classification of the FFPE and frozen slides of the test set. (a) Subtype classification AUCs of the FFPE slides. (b) Subtype classification AUCs of the frozen slides. (c) and (d) show scatter plots of AUC for frozen samples and FFPE samples for each tumor-type using (c) macro-average and (d) micro-average AUC values. Frozen and FFPE values are highly correlated for both macro- (r=0.87) and micro- (r=0.78) averages. Sample sizes are provided in Figure 3a.

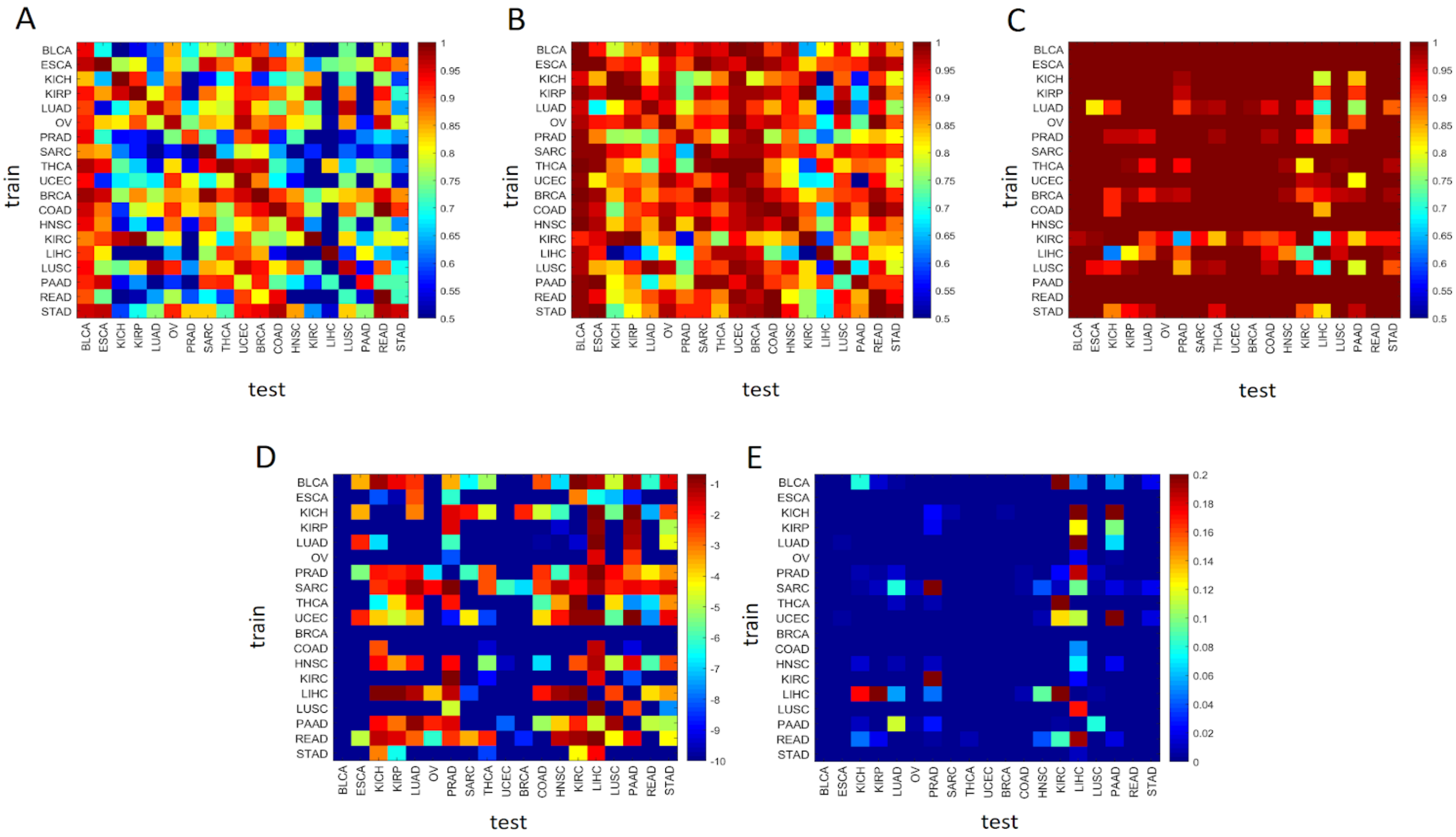

Supplementary Figure 5. Confidence interval of AUCs for tumor normal and cross classification models. The lower bound of the CI, mean AUC, and upper bound of the CI are presented in subfigures (A), (B), and (C), respectively. Out of the 19*19=361 cross-classification models, the lower bound on the CI of 164 models is above 80%, suggesting the presence of strong common morphological features across various cancer types. Subfigures (D) and (E) provide the log10 and adjusted p-values of the hypothesis tests for AUCs being larger than 0.5 (null AUC=0.5, alternative AUC>0.5). 330 out the 361 classification models are significant (have AUC>0.5) while bounding FDR by 5%.

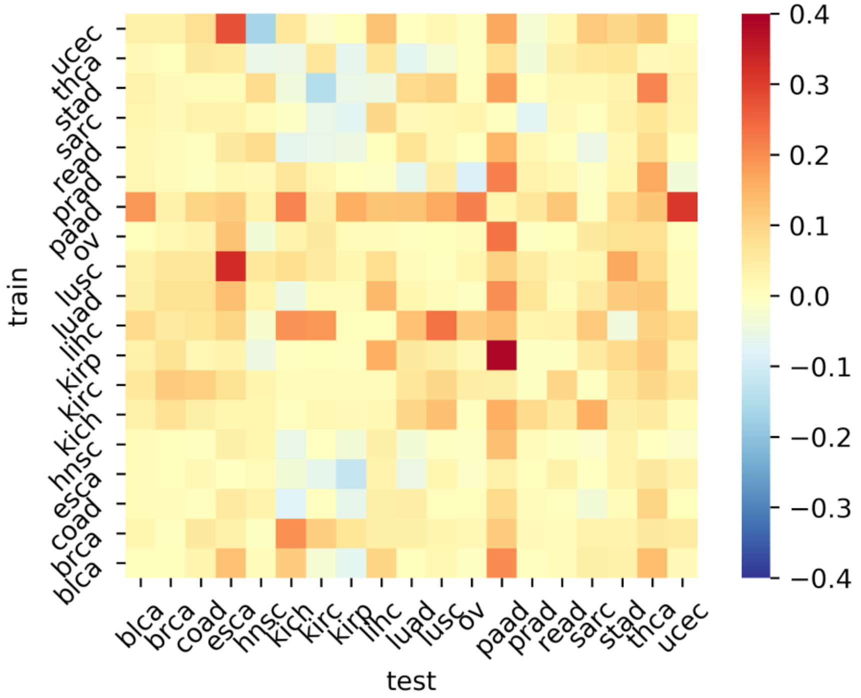

Supplementary Figure 6. The difference between the self- and cross-classification AUCs of the proposed architecture of Figure 1a and the original inception V3 network. Positive values denote a higher test AUC for the proposed network of Figure 1a.

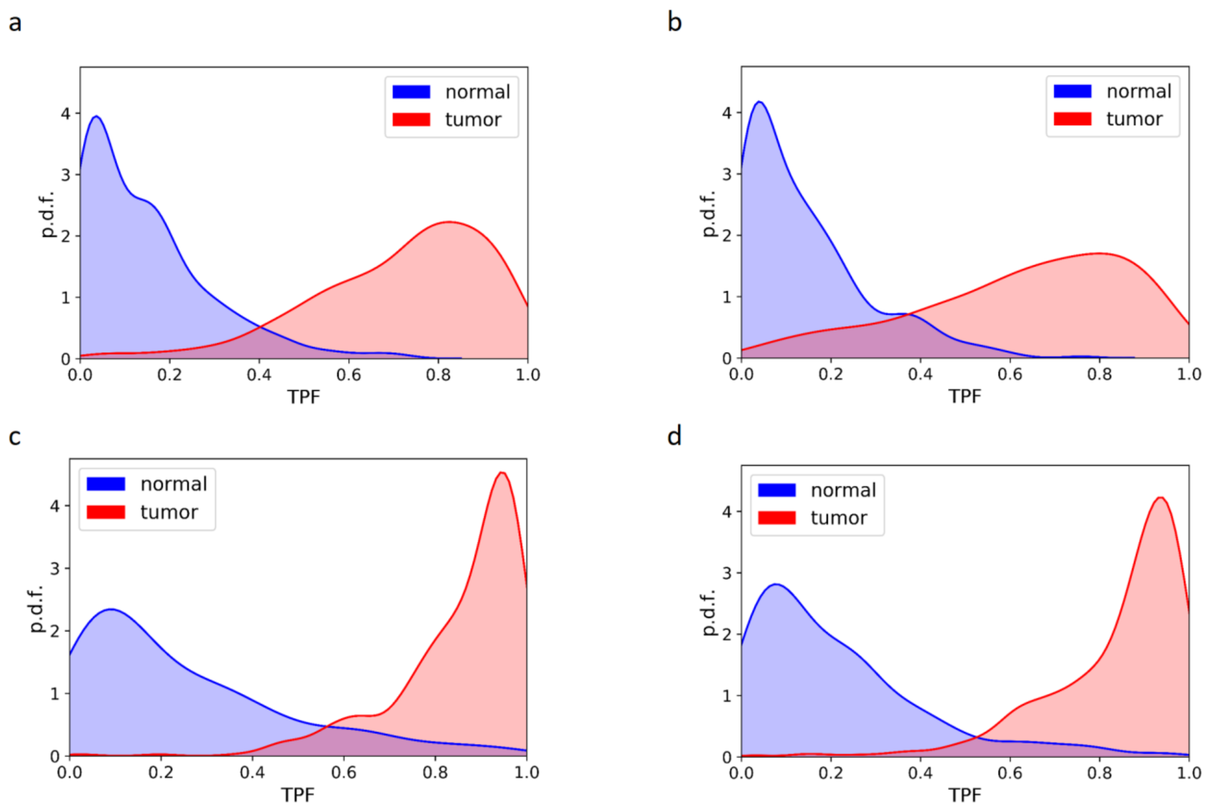

Supplementary Figure 7. The probability density function of TPF for tumor and normal validation slides. (a) TCGA-LUAD classifier applied to CPTAC-LUAD data. (b) TCGA-LUSC classifier applied to CPTAC-LUAD data. (c) TCGA-LUAD classifier applied to CPTAC-LUSC data. (d) TCGA-LUSC classifier applied to CPTAC-LUSC data.

a

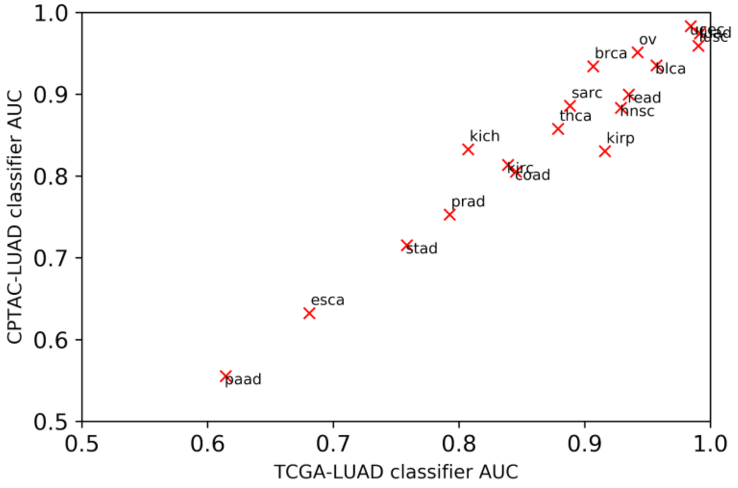

b

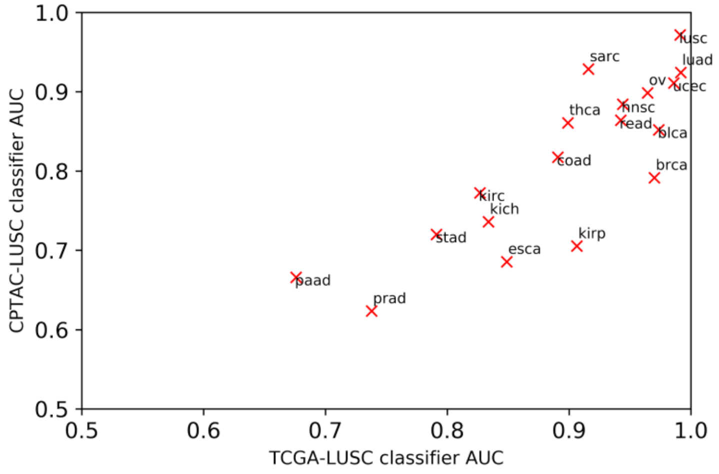

Supplementary Figure 8. Cross-classification AUC comparison of TCGA-trained and CPTAC-trained LUAD and LUSC classifiers on TCGA test sets for 19 cancers. (a) Cross-classification AUCs of TCGA-trained and CPTAC-trained LUAD classifiers (r=0.98). (b) Cross-classification AUCs of TCGA-trained and CPTAC-trained LUSC classifiers (r=0.90). TCGA test set sizes are provided in Figure 2a.

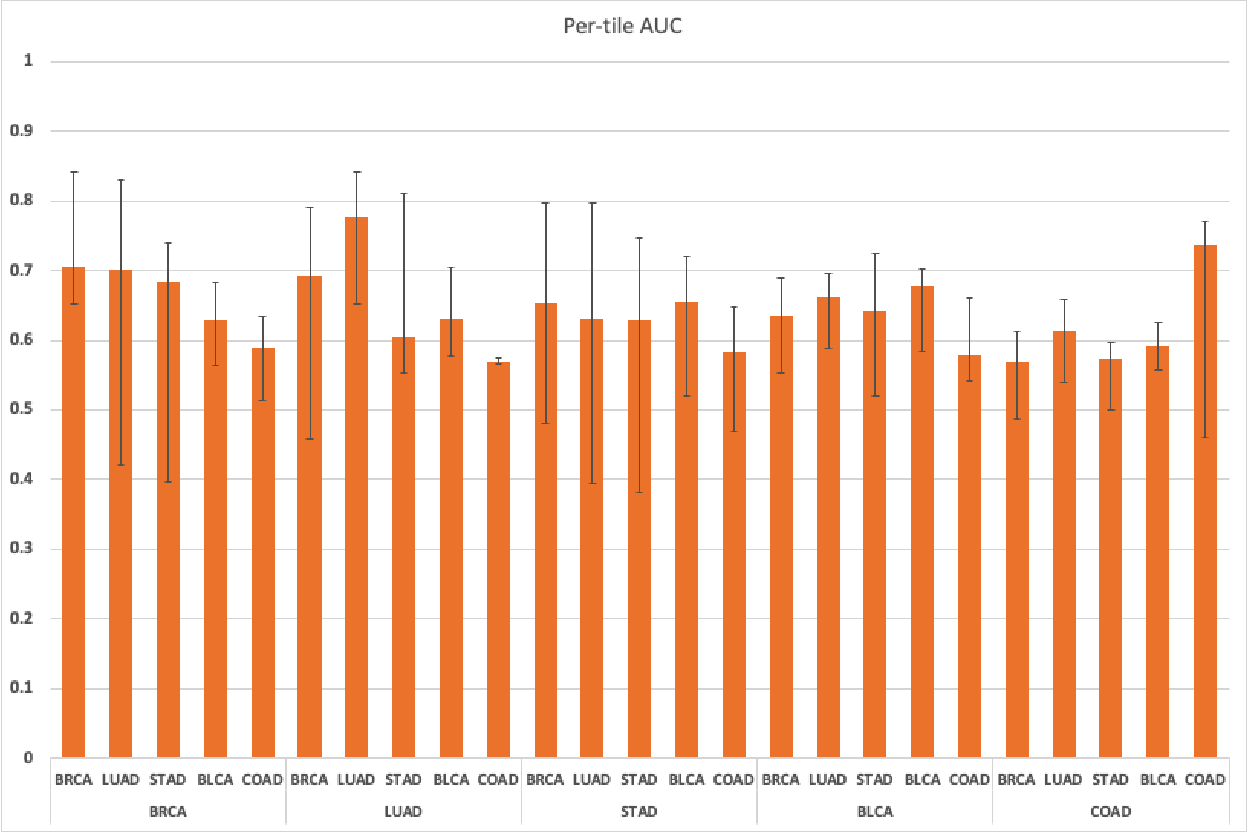

Supplementary Figure 9. Per-tile AUC values for TP53 mutational status cross-classification experiments along with their confidence intervals. Bars denote the lower and upper bounds of the 95% CI. Sample sizes are provided in Table 1.

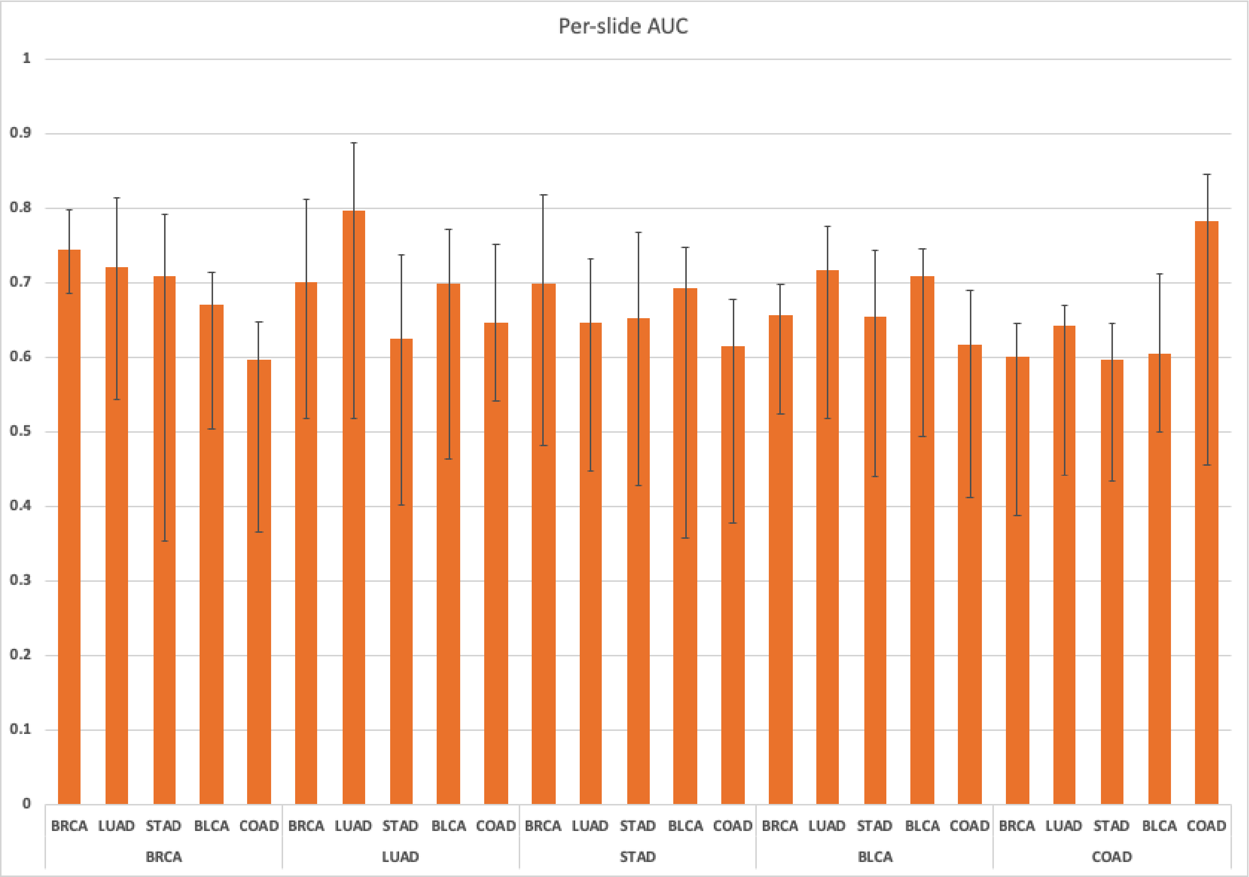

Supplementary Figure 10. Per-slide AUC values for TP53 mutational status cross-classification experiments along with their confidence intervals. Bars denote the lower and upper bounds of the 95% CI. Sample sizes are provided in Table 1.

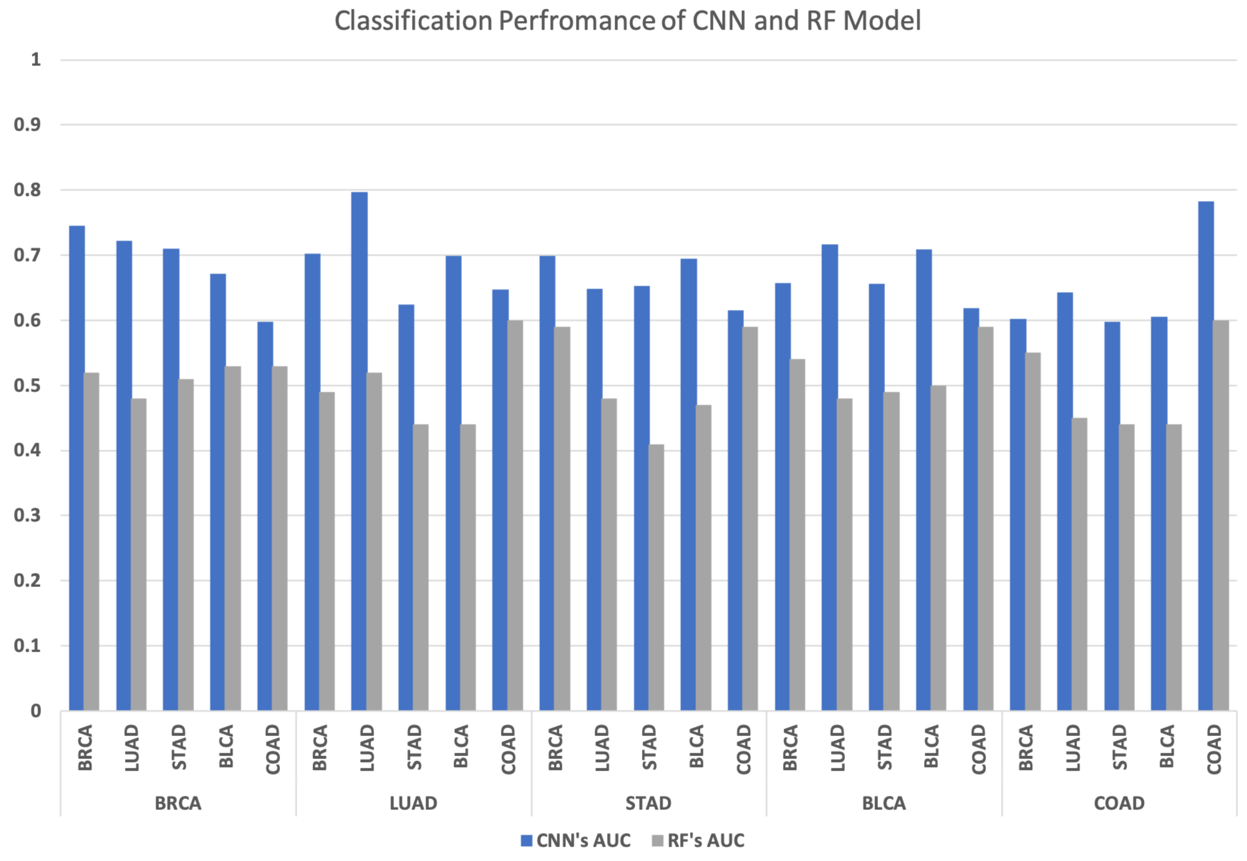

Supplementary Figure 11. TP53 mutational classification performance comparison between CNN model and Random Forest model trained on tumor purity and stage. To determine whether the CNN model uses information more sophisticated than tumor purity and stage to predict TP53 mutational status, we compared its performance to a random forest model. The random forest model was trained to predict TP53 status using only tumor purity and stage for each of the five cancer sets. Training was performed at slide level. The corresponding AUC self- and cross-classification values are shown below, with the CNN-based AUCs shown for comparison. The AUC values from the Random Forest model are lower than the AUCs from the CNN model in all cases. Sample sizes are provided in Table 1.

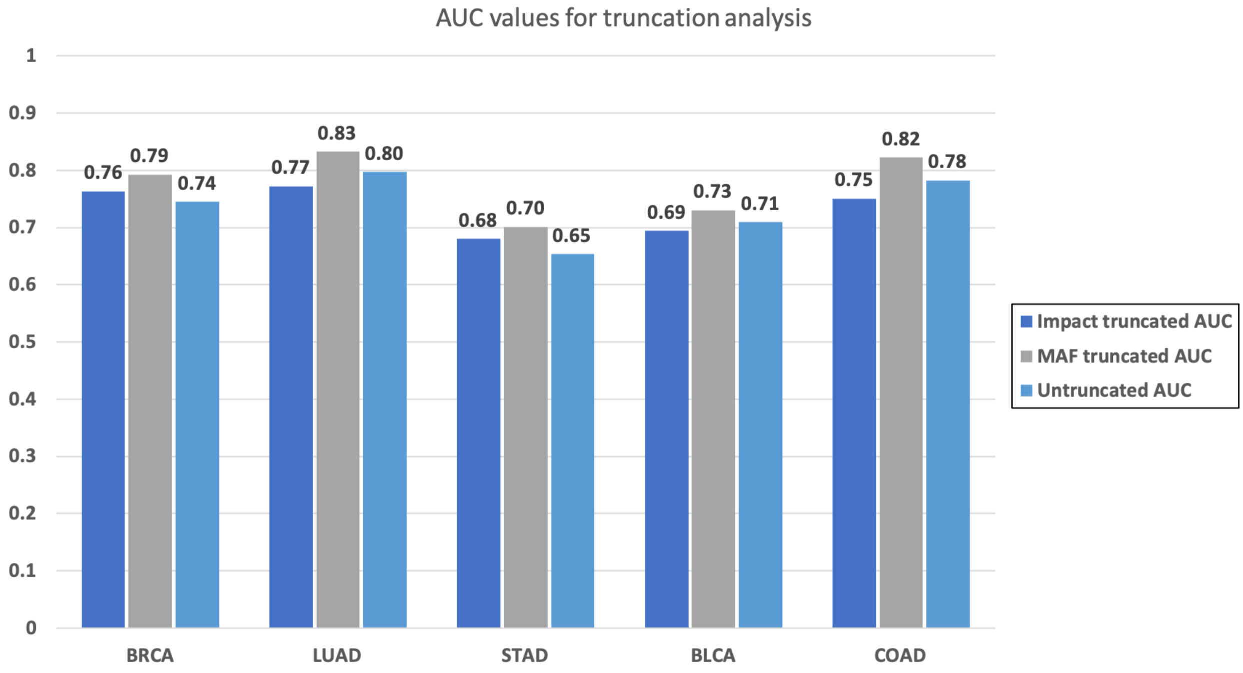

Supplementary Figure 12. TP53 mutational classification performance as a function of allele frequency and mutational impact. We considered classification performance when using an additional minimum threshold for TP53 mutation frequency which should favor cases where the mutation is ubiquitous throughout the tumor. We tested our trained CNN model within each cancer type, with a requirement of high minor allele frequency (MAF > 0.25).Our model has increased AUC on such cases in all cancer types. We also analyzed samples based on a strict IMPACT metric (requiring IMPACT=HIGH). Truncating the sample by this IMPACT constraint does not lead to a systematic improvement in AUC, as different cancer types show varying effects. Test set is similar in size to the set described in Table 1, except positive class labels are now determined using additional constraints.

a

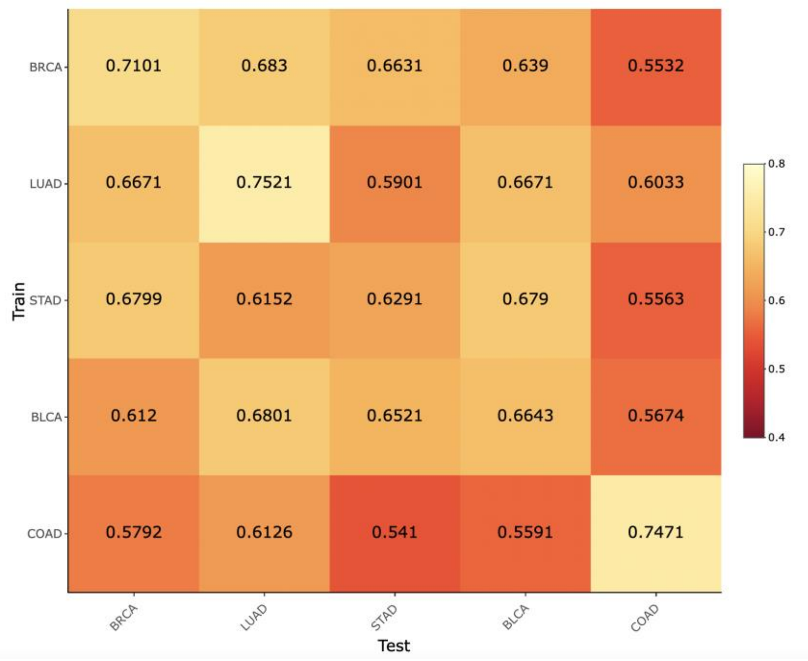

b

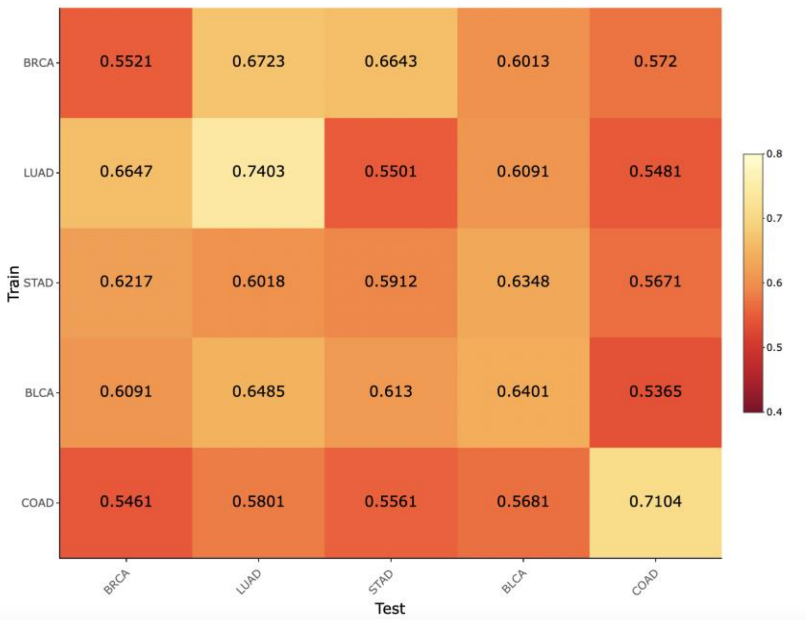

Supplementary Figure 13. F1 scores of slide-level and tile-level TP53 mutational classification performance. a) Cross- and self- classification F1-score values from balanced deep learning models (with 95% CIs) are given (a) per-slide and (b) and per-tile.

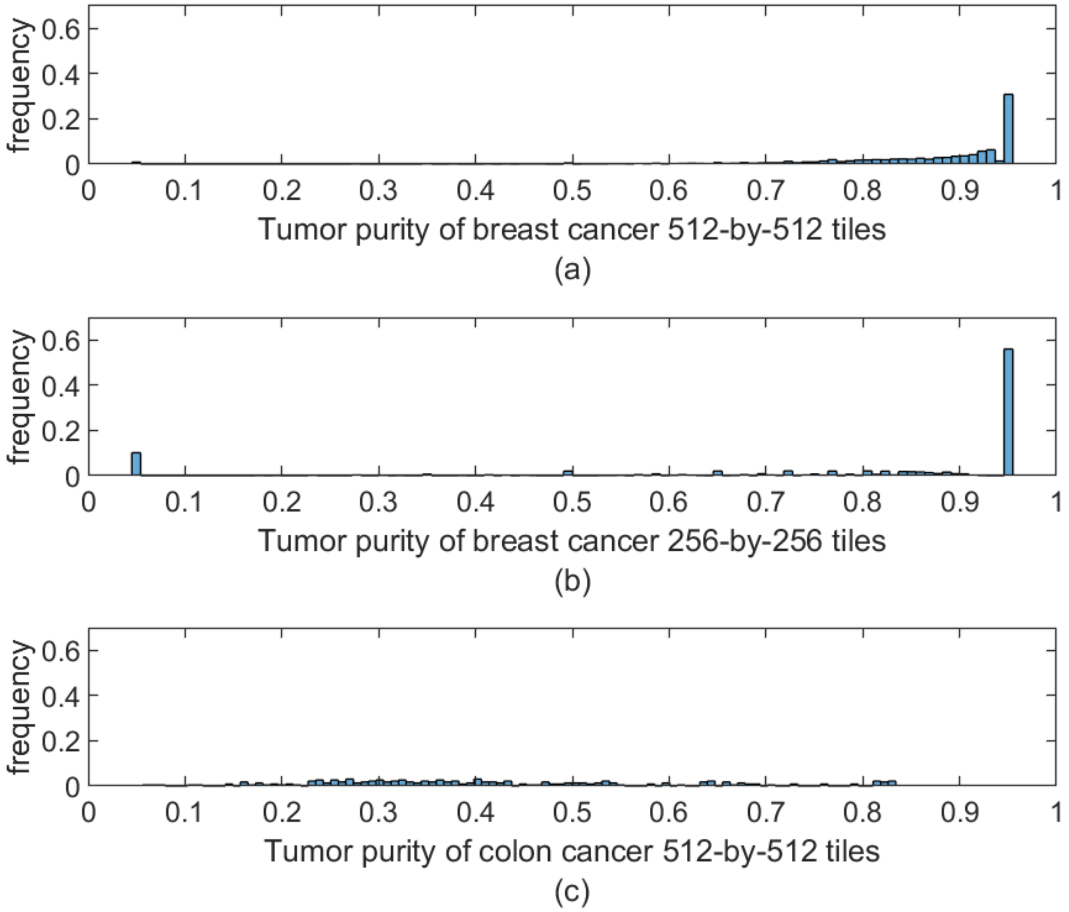

Supplementary Figure 14. Purity histogram of breast and colon cancer ROIs. Distribution of regularized TPF across the tiles of breast and colon cancer ROIs. While the breast cancer dataset used for training is mostly comprised of tiles with large TPFs, the colon cancer validation set has a more spread TPF.
