# Supplementary material for: Deep learning-based cross-classifications reveal conserved spatial behaviors within tumor histological images
Source: Nat Commun. 2020 Dec 11;11:6367. doi: 10.1038/s41467-020-20030-5 (PMC7733499; doi:10.1038/s41467-020-20030-5)
Supplement: Supplementary file 8 — Description of Additional Supplementary Files [file 41467_2020_20030_MOESM8_ESM.pdf]

Title: Supplementary Data 1:
Description: AUC and its corresponding CI for cancer subtype classification at the slide level. P-values are based on one versus all comparisons, null expectation AUC=0.5.

Title: Supplementary Data 2:
Description: Cross-classifier correlations for the 3 test tissues with the maximal correlations.

Title: Supplementary Data 3:
Description: The number of test tiles of each cancer used to measure prediction accuracy in Figure 2b.

Title: Supplementary Data 4:
Description: Raw and adjusted p-values of Figure 2e computing the correlations between TPF and tumor purity.

Title: Supplementary Data 5:
Description: Patient level stratification test set sizes reported in number of test slides.
